# Tubal ectopic molar pregnancy: A case report

**DOI:** 10.18502/ijrm.v22i5.16441

**Published:** 2024-07-08

**Authors:** Mojgan Hajisafari Tafti, Sajad Zare Garizi, Fatemeh Mazidi

**Affiliations:** ^1^Department of Obstetrics and Gynecology, School of Medicine, Shahid Sadoughi University of Medical Sciences, Yazd, Iran.; ^2^Research and Clinical Center for Infertility, Yazd Reproductive Sciences Institute, Shahid Sadoughi University of Medical Sciences, Yazd, Iran.; ^3^Student Research Committee, Shahid Sadoughi University of Medical Sciences, Yazd, Iran.

**Keywords:** Ectopic molar pregnancy, Ectopic pregnancy, Hydatidiform mole.

## Abstract

**Background:**

Ectopic molar pregnancy (EMP) is a rare form of gestational trophoblastic disease that occurs when a hydatidiform mole implants outside the uterus.

**Case Presentation:**

We describe a 35-yr-old woman with mild abdominal pain, delayed menstruation for 2 months, and high beta-human chorionic gonadotropin levels. Sonography revealed a heterogeneous hyperechoic mass in the left adnexa and fluid in the endometrial cavity, suggestive of a tubal EMP. She underwent endometrial curettage and left salpingectomy. Pathology confirmed the diagnosis of invasive hydatidiform mole/left tubal EMP. The case recovered well and had no complications.

**Conclusion:**

This case highlights the need for early diagnosis and multidisciplinary treatment of EMP to avoid serious consequences from persistent trophoblastic tumors.

## 1. Introduction

Gestational trophoblastic disease (GTD) is a group of disorders that involve abnormal growth of placental tissue during or after pregnancy. GTD can be benign or malignant and can affect the uterus or other sites (1). The types of GTD are hydatidiform mole (HM), choriocarcinoma, placental site trophoblastic tumor, and epithelioid trophoblastic tumor (2–4).

HMs are the most common type of GTD, affecting about 1 in 500–1000 pregnancies in Western countries (5). A complete mole results from fertilizing an empty egg with one or 2 sperm, producing a mass of trophoblastic tissue without fetal parts. A partial mole results from fertilizing a normal egg by 2 sperm, producing a mixture of trophoblastic and fetal tissue with a triploid karyotype (3). An invasive mole is a type of GTD that develops from an HM that invades the myometrium or other structures. It is characterized by both trophoblastic hyperplasia and villous myometrial invasion (penetration of chorionic villi into the uterine wall). Molar pregnancy can sometimes lead to invasive HM, which is a rare condition that affects 1 in every 15,000 pregnancies (6). Invasive mole was first documented in Madagascar in 1965, and another case was identified in 2018 (7, 8).

Ectopic molar pregnancy (EMP) is a rare form of GTD that occurs when an HM grows outside the uterus, typically in the fallopian tube. EMP affects 1 in every 20,000–100,000 pregnancies and represents less than 4% of all GTD cases (2, 9). According to a 15-yr analysis of Sheffield Trophoblastic Disease Center cases, EMP is rare, occurring in 1.5 per million deliveries in the UK (10).

This study aims to delineate a case of invasive HM in the left fallopian tube and to evaluate the clinical, diagnostic, and therapeutic dimensions associated with this condition.

## 2. Case Presentation

The present case was a 35-yr-old woman, gravida 5, living 2, para 2, abortion 2, who had visited a gynecologist due to mild abdominal pain, retard menstruation for 2 months, and increased level of beta human chorionic gonadotropin ( β -hCG, 65481 IU/L). After performing sonography with the diagnosis of ectopic pregnancy, the case was admitted to the obstetrics and gynecology department of Shahid Sadoughi hospital in Yazd, Iran for diagnostic and therapeutic procedures. According to the last menstrual period, the case's gestational age was 8 wk + 4 days. The case had a history of hypothyroidism with a daily intake of levothyroxine 100 μ g tablets, a history of one curettage due to abortion, and laparoscopy for cholecystectomy. The case had 2 normal vaginal deliveries.

She underwent a general physical examination which had no pathological findings except a mild tenderness in the left lower quadrant of the abdomen; blood pressure was 100/75, pulse 85, and a temperature of 37 C. The chest was clear, and the electrocardiography was normal. Also, there was no tenderness in the abdomen.

**Figure 1:**
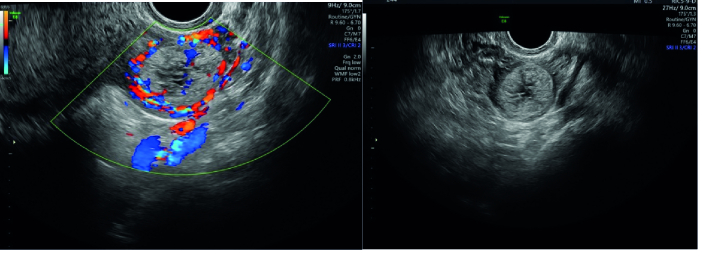
Transvaginal ultrasonography image, molar ectopic pregnancy in left adnexa with snow storm view.

In the initial sonography, the case's uterus size was 90 × 50 mm. In the endometrial cavity, fluid accumulation was observed with an irregular shape of about 22 mm, surrounded by a thick area suspicious of a gestational sac without a fetal pole and decidua. However, in the left para ovarian adnexa, a brief heterogeneous hyperechoic mass with a snowstorm appearance and a cystic center with a diameter of 29 × 38 mm was observed with vascular flow (Figure 1).

The laboratory results showed hemoglobin 12.8 g/dL, hematocrit 34.1%, white blood cells 9900/ μ L, red blood cells 4.00 mil/ μ L, platelets 210,000/ μ L, and quantitative β -hCG level 73224 mL. Considering the high and ascending trend of β -hCG titer and sonographic evidence, we suspected EMP due to changes in the endometrial cavity.

**Figure 2:**
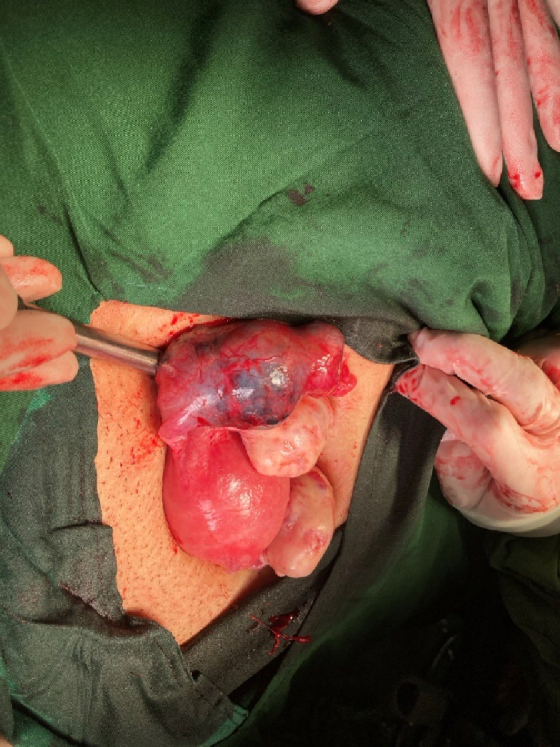
Intraoperative view of an invasive hydatidiform mole in the left fallopian tube.

**Figure 3:**
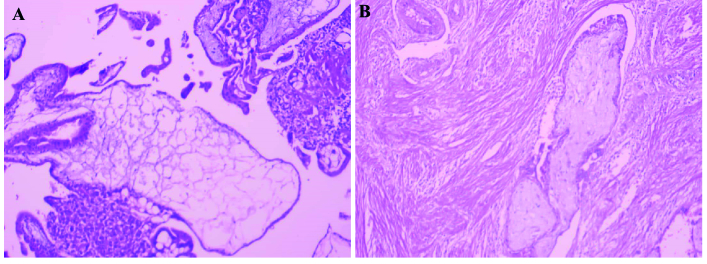
Microscopic pathology image. A) Molar chorionic villi with prominent trophoblastic hyperplasia. B) Intravascular invasion of molar chorionic villi.

She was given general anesthesia, followed by endometrial curettage, the tissue was then sent to the pathology based on the sonography results. Then, in the supine position, the abdomen was opened by a transverse mini-laparotomy method, where a 4 cm mass suspected of EP was seen in the left tube. This involved the entire tube, except the fimbriae, resulting in left salpingectomy, the sample was then sent to the pathology (Figure 2). The histopathology results confirmed the diagnosis of invasive HM/left tubal ectopic pregnancy during her follow up (Figure 3).

She had a good general condition, stable signs, and was discharged 2 days after admission. After discharge, β -hCG level was measured weekly and the first negative result was obtained after 4 wk. Then, the β -hCG level was measured monthly until 6 months, which was negative. Therefore, the possibility of recurrence and metastasis has been ruled out for the patient. Additionally, the metastasis workup including chest X-ray graphy and CT scan of the abdomen and pelvis has been ordered for the patient. Unfortunately, she refused to perform.

### Ethical considerations

A written informed consent was obtained to use the medical records for any research purpose.

## 3. Discussion

HM is a form of GTD that arises from abnormal growth of villous trophoblast due to a chromosomal error during gametogenesis and fertilization, related to placental defects. HM has 2 types: partial HM, which has a mixture of molar and normal placental villi, and complete HM, which has only molar villi (11).

Molar pregnancy is more likely to occur in women who have a low intake of vitamin A and animal fat, are older than 40 yr, or have had repeated miscarriages. The causes of ectopic pregnancy are well established (11, 12).

EMP is a rare condition that occurs when an HM develops outside the uterus, usually in the fallopian tube. The first case of EMP was reported in 1871, and since then, only 132 cases of tubal ectopic pregnancy have been confirmed in the world literature until 2005 (13). Gillespie and co-authors documented the initial UK series, comprising 31 cases of ectopic molar gestation. They observed a rarity in ectopic GTD, with a UK incidence of approximately 1.5 per 1,000,000 births (10). To the best of our knowledge, this marks the second case of tubal invasive mole reported in the Iranian literature. Najib and colleagues reported a case of a 26-yr-old woman, nulli gravid, who had her first complete molar pregnancy inside the uterus. She underwent suction curettage but developed gestational trophoblastic neoplasm, and received chemotherapy. During chemotherapy, she experienced severe abdominal pain and had a laparotomy, where an EMP was also found in her fallopian tube. A salpingectomy was done and her hCG levels were monitored. She was diagnosed again with postmolar heterotopic gestational trophoblastic neoplasm because of abnormally low hCG levels and got multiple doses of methotrexate, but did not respond. Therefore, she received 5 courses of actinomycin-D as a treatment and had her monthly follow-up for her hCG level (14).

Mbarki and colleagues reported 2 cases of tubal ectopic pregnancy with molar components. The first case was a 32-yr-old woman who had a partial molar pregnancy in the left tube. She presented with acute abdominal pain, mild bleeding for 10 hr, and a left adnexal mass with a live embryo at 6 wk of gestation. She had a laparoscopic left salpingectomy. The second case was a 37-yr-old woman who had a complete molar pregnancy in the left tube that ruptured. She had severe pelvic pain and 7 wk of amenorrhea. She also had a laparoscopic left salpingectomy (11).

Invasive mole is a rare condition that usually develops from an HM within 6 months of its presentation and diagnosis when it comes to EMP. It can be difficult to diagnose, as it resembles normal tubal ectopic pregnancy clinically (11, 15). Rahaoui and colleagues reported 2 cases of invasive mole that occurred 3 months and 3 yr after the removal of the complete mole (16).

Most reported cases of invasive moles involve the uterus and cause vaginal bleeding and severe abdominal pain that necessitate medical attention. In contrast, the case in the present study had a good general condition and mild abdominal pain and sought medical care because of delayed menstruation. Most reported cases of invasive moles involve the uterus and cause vaginal bleeding and severe abdominal pain that necessitate medical attention (6, 15, 17). In contrast, the case in the present study had a good general condition and mild abdominal pain and sought medical care because of delayed menstruation. Therefore, it can be said that intrauterine molar pregnancy is more symptomatic and easier to diagnose.

Ultrasound images commonly depict invasive mole, implantation site tumors, and choriocarcinomas as heterogeneous, hyperechoic solid masses with cystic vascular spaces within the myometrium. Ultrasound is one of the best diagnostic methods before pathology. In this case, we had most of the sonographic criteria for molar intrauterine pregnancy. So, special attention to sonographic evidence in EMP along with the very high level of β -hCG is necessary (15).

Molar pregnancy requires strict morphologic criteria for diagnosis. To avoid over diagnosing tubal HM, some authors recommended combining histological features and DNA flow cytometry to assess cases of possible molar tubal ectopic pregnancy (18, 19). EMP requires surgical removal of the conceptus, preferably by laparoscopy, and complete excision of the trophoblast. The prognosis of EMP is similar to other types of GTD. Hence, molar pregnancy has the potential to result in persistent trophoblastic disease and malignant transformation, similar to intrauterine molar pregnancies. Postoperative follow-up is crucial, requiring ongoing monitoring of β -hCG until normalization, along with appropriate counseling and contraception. The reported cases exhibited a favorable clinical progression with no complications (6, 11).

## 4. Conclusion

EMP is infrequent. The definitive confirmation and determination of molar pregnancy type relies on pathological examination of resected tubal specimens. Timely identification is paramount to averting life-threatening complications, especially in women of childbearing age, where a complete mole might progress to a persistent trophoblastic tumor. The diagnosis is validated through histological examination, and a multidisciplinary approach. Radiologic assessment especially in cases of EMP, enhances the prognosis for these rare conditions.

##  Data availability

The data that support the findings of this study are available from the corresponding author, Mojgan Hajisafari Tafti, upon reasonable request.

##  Author contributions

ZG.S. and M.F. designed the study. HT.M. and M.F. identified and collected the data. ZG.S. and M.F. wrote and reviewed the paper. HT.M. critically reviewed the paper. All authors approved the final version of the paper and took responsibility for its content.

##  Conflict of Interest

The authors declare that there is no conflict of interest.
